# Selection of endogenous control genes for normalising gene expression data derived from formalin-fixed paraffin-embedded tumour tissue

**DOI:** 10.1038/s41598-020-74380-7

**Published:** 2020-10-14

**Authors:** Tim A. D. Smith, Omneya A. AbdelKarem, Joely J. Irlam-Jones, Brian Lane, Helen Valentine, Becky A. S. Bibby, Helen Denley, Ananya Choudhury, Catharine M. L. West

**Affiliations:** 1 Translational Radiobiology Group, Division of Cancer Sciences, University of Manchester, Manchester Academic Health Centre, Christie Hospital NHS Found Trust, Manchester, M20 4BX UK; 2 Medical Research Institute, Alexandria University, 165 El-Horreya Avenue, El-Hadra, Alexandria, Egypt; 3 Pathology Centre, Shrewsbury and Telford NHS Trust, Royal Shrewsbury Hospital, Shrewsbury, SY3 8XQ UK

**Keywords:** Surgical oncology, Cancer genomics, Gene expression

## Abstract

Quantitative real time polymerase chain reaction (qPCR) data are normalised using endogenous control genes. We aimed to: (1) demonstrate a pathway to identify endogenous control genes for qPCR analysis of formalin-fixed paraffin-embedded (FFPE) tissue using bladder cancer as an exemplar; and (2) examine the influence of probe length and sample age on PCR amplification and co-expression of candidate genes on apparent expression stability. RNA was extracted from prospective and retrospective samples and subject to qPCR using TaqMan human endogenous control arrays or single tube assays. Gene stability ranking was assessed using coefficient of variation (CoV), GeNorm and NormFinder. Co-expressed genes were identified from The Cancer Genome Atlas (TCGA) using the on-line gene regression analysis tool GRACE. Cycle threshold (Ct) values were lower for prospective (19.49 ± 2.53) vs retrospective (23.8 ± 3.32) tissues (*p* < 0.001) and shorter *vs* longer probes. Co-expressed genes ranked as the most stable genes in the TCGA cohort by GeNorm when analysed together but ranked lower when analysed individually omitting co-expressed genes indicating bias. Stability values were < 1.5 for the 20 candidate genes in the prospective cohort. As they consistently ranked in the top ten by CoV, GeNorm and Normfinder, *UBC, RPLP0, HMBS, GUSB, and TBP* are the most suitable endogenous control genes for bladder cancer qPCR.

## Introduction

Bladder cancer is a major cause of morbidity and mortality in the UK. Data from Cancer Research UK showed that it is the tenth most common cancer in the UK, accounting for 3% of all new cancer cases^1^. There are multiple therapeutic options for bladder cancer, which highlights the importance of developing biomarkers for personalising treatment^2^. Emerging transcriptomic signatures can be progressed towards clinical application using different platforms including quantitative real time polymerase chain reaction (qPCR).

qPCR is a sensitive, affordable and efficient method for measuring gene expression in tissue samples including formalin fixed paraffin embedded tissue (FFPE). RNA from FFPE is generally of poor quality^3^. Formalin fixation results in cross-linking of RNA with other macromolecules including DNA and protein which when dissociated during RNA purification results in fragmentation and reduction in yield of probable material. Pre-amplification of cDNA from these samples is necessary to obtain quantifiable data^4^. Samples of cDNA can then be subject to qPCR. Most qPCR data measure relative gene expression via normalization with endogenous control genes (also known as reference or housekeeping genes). Genes used widely in the past can be affected by tissue type and experimental conditions^5–7^, and it is important to identify genes with constitutive and invariant expression for the samples of interest.

Some studies used multiple endogenous control genes that include several co-expressed genes including the ribosomal protein large (RPL) family of proteins^8,9^. Where co-expressed genes are present within a candidate gene panel their mutual influence on apparent stability requires consideration. Genes with similar functions tend to exhibit similar gene expression patterns^10^. Gene co-expression can be checked using the on-line tool Genomic Regression Analysis of Coordinated Expression (GRACE) which correlates (Spearman) the expression of a gene with all other genes within TCGA. Vandesompele et al.^11^ propose testing a panel of candidate reference genes on a representative number of relevant samples to identify the most stable and optimal number of genes. Test data generated are subject to stability assessment algorithms, the two most commonly used are GeNorm^11^ and NormFinder^12^. These algorithms rank genes in order of stability and in the case of GeNorm select the two-gene combination that provides the most stable normalization. GeNorm is considered the optimal algorithm for studies with small sample numbers^12^ but over-rates the stability of co-expressed genes in the candidate panel.

Gene expression data are highly dependent on platform^13^ so endogenous control gene selection is carried out on the platform of choice. To facilitate selection of control genes, TaqMan endogenous control array cards are available with 16 candidate genes. These genes have been used for normalization in human tissue gene expression studies including bladder^14^, thyroid^15^, hepatocellular^16^, breast^9^, gastric^17^, cervical^18^, endometrial^19^, non-small cell lung^20^, kidney^21^ and prostate^22^ cancer. Whilst bioinformatic interrogation of TCGA provides a useful verification of gene expression stability it is unsuitable for endogenous control gene selection for TaqMan Array cards as the TCGA database is derived using RNA sequencing.

The primary aim of this work is to facilitate selection of endogenous control genes for the Taqman qPCR gene expression platform for studies of prospective FFPE cancer tissue using bladder cancer as an exemplar. The secondary aim is to evaluate the influence of some covariables including probe length on reverse transcription efficiency and co-expression on stability ranking.

## Materials and methods

### Patient samples

Pre-treatment FFPE grade 3 MIBC samples were available from a prospective (n = 12) and retrospective (n = 16) patient cohort. Samples were obtained via the Manchester Cancer Research Centre Biobank under research tissue bank ethics (Ref: 18/NW/0092). The cases were graded by an experienced subspecialist Uropathologist (HD). Mean (range) block age was 6 (3–8) months for the prospective cohort and 15 (14–17) years for the retrospective cohort. Two 10 µm sections for RNA extraction and a 4 µm section for histological verification of tumour cellularity (> 30%) were obtained from each block. RNA was extracted from the two 10 µm sections using the Roche High Pure FFPET RNA isolation kit. TCGA bladder cancer RNA-seq data (n = 401) were also analysed.

### TLDA cards and single tube assays

Table 1 lists the endogenous control genes tested along with the probes and their amplicon length. Sixteen genes were on the endogenous control TLDA cards and single tube assays were set up for succinate dehydrogenase complex flavoprotein subunit A (*SDHA*) a gene demonstrating particularly low variability in bladder cancer cells^9^. Single tube assays were also run for *RPL11*, *RPL24* and *GNB2L1* gene to examine the biasing effect of co-expression on gene stability. To investigate the effect of probe size on Ct values two different probes were selected for *RPL11*, *RPL24* and *GNB2L1*.

**Table 1:** Candidate endogenous control genes with the Thermo Fisher gene probe, amplicon length and intra-assay reliability.

Candidate gene	Gene probe	Amplicon size (bp)	Intra-sample Ct SD
*18S rRNA*	18S-Hs99999901_s1	187	0.250
*ACTB*	ACTB-Hs99999903_m1	171	0.370
*B2M*	B2M-Hs99999907_m1	75	0.083
*GAPDH*	GAPDH-Hs99999905_m1	122	0.163
*GUSB*	GUSB-Hs99999908_m1	81	0.108
*HMBS*	HMBS-Hs00609297_m1	64	0.090
*HPRT1*	HPRT1-Hs99999909_m1	100	0.232
*IPO8*	IPO8-Hs00183533_m1	71	0.247
*PGK1*	PGK1-Hs99999906_m1	75	0.323
*POLR2A*	POLR2A-Hs00172187_m1	61	0.150
*PPIA*	PPIA-Hs99999904_m1	98	0.150
*RPLP0*	RPLP0-Hs99999902_m1	105	0.101
*TBP*	TBP-Hs99999910_m1	127	0.136
*TFRC*	TFRC-Hs99999911_m1	105	0.095
*UBC*	UBC-Hs00824723_m1	71	0.106
*YWHAZ*	YWHAZ-Hs00237047_m1	70	0.084
*SDHA*	Hs00188166_m1	70	0.065
*RPL11*	Hs00831112_s1	142	0.129
*RPL11*	Hs04183527_g1	106	0.117
*RPL24*	Hs02338570_gH	156	0.091
*RPL24*	Hs07291664_gH	86	0.058
*GNB2L1*	Hs00914568_g1	75	0.095
*GNB2L1*	Hs00272002_m1	66	0.046

Reliability was measured as intra-sample standard deviation (SD) of cycle threshold (Ct) values assayed in triplicate. Each probe was assayed in 28 samples and the mean of the SD values calculated.

### RNA extraction, quantification and reverse transcription

RNA was extracted using the Roche High Pure FFPET RNA isolation kit from two 10 µm sections. The detailed steps of extraction were performed according to manufacturer’s recommendations. RNA quantification and purity were determined on a NanoDrop UV–Vis Spectrophotometer (Thermo Fisher Scientific Poole UK) and a Qubit fluorometer (Invitrogen Paisley UK). Reverse transcription and pre-amplification steps were carried out on a 2720 thermal cycler (Applied Biosystems UK). qPCR was carried out on a Quantstudio 12K (Applied Biosystems UK). Complementary DNA (cDNA) was generated using a high capacity RNA-to-cDNA kit (Life Technologies Ltd UK). One sample of cDNA was subject to pre-amplification using a custom preamp pool mix consisting of gene expression assay corresponding with genes present on the TaqMan human endogenous control card array (Applied Biosystems®). A further sample of cDNA underwent pre-amplification using a preamp pool mix prepared by mixing single tube assay (Thermo Fisher Scientific UK) components for the panel of candidate genes not present on the endogenous control card array. A preamp TaqMan Fast Advanced Master Mix (Thermo Fisher Scientific UK) was used for both samples. The pre-amplification step was carried out for 14 cycles on a 2720 thermal cycler.

### qPCR

Samples pre-amplified using the control array primer pool were mixed with Fast Mastermix (2X) and loaded onto the endogenous control plate and subject to qPCR on a Quantstudio12 (Applied Biosystems). Samples pre-amplified using the pooled single assay primer pool were loaded into 96 well plates preloaded with individual gene probes and Fast Mastermix (2x) and subject to qPCR on a Quantstudio12 using the fast cycle option.

### Data analysis

TCGA was accessed using the Firebrowse portal selecting RSEM normalised RNAseq bladder cancer. Each gene was examined for co-expression with other genes within the candidate panel using the on-line tool GRACE. Coefficient of variation (CoV) values for the expression of each gene were calculated and significant differences between mean values determined using the Student t-test.

GeNorm (https://genorm.cmgg.be/) and Normfinder (NormFinder software—moma.dk) algorithms were used to determine the most stable genes from the list of candidates. The software packages were used as excel add-ons. To determine the biasing effect of co-expressed genes on apparent gene expression stability using GeNorm, the analyses were carried out with all candidate genes and repeated after removing 3 of the 4 co-expressed genes.

Statistical significance between means was determined using the Mann Whitney U test calculator (Wilcoxon rank-sum) non-parametric test.

## Results

### RNA yield, quality and expression reliability

The mean (range) yields were 291 (50–560) ng/µl for the 12 prospective and 251 (64–425) ng/µl for the 16 retrospective samples. The mean (range) RNA quality ratios were 1.90 (1.93–2.19) for 260/280 and 2.00 (1.72–2.12) for 260/230 for the prospective samples. Respective values for retrospective samples were 1.88 (1.56–1.98) and 1.73 (1.53–2.00). Gene expression was determined in triplicate and the intra-sample standard deviation (SD) of the cycle threshold (Ct) values (number of cycles required for the fluorescent signal to cross a threshold) calculated for the 28 samples. Table 1 lists the mean SD for each of the 23 candidate endogenous control gene probes. The mean ± SD of the SD of the Ct values for triplicate assays for 16 endogenous control genes assayed in 28 samples on TaqMan arrays was 0.153 ± 0.071 (n = 448 gene-probe/sample combinations; range 0.079 to 0.37). For the 7 single tube assays the mean SD for the Ct values were 0.082 ± 0.022 (n = 196 gene-probe/sample combinations; range 0.046 to 0.129). To investigate inter-assay reliability three samples were assayed by qPCR on two separate TaqMan arrays/96 well plates run on two different days. The mean ± SD of the SD of the Ct values for each gene/sample (n = 48) was 0.21 ± 0.04 for samples loaded onto TLDA cards and 0.07 ± 0.039 for the single tube assays loaded into 96 well plates.

### Effects of FFPE block age and gene probe length

**Figure 1:**
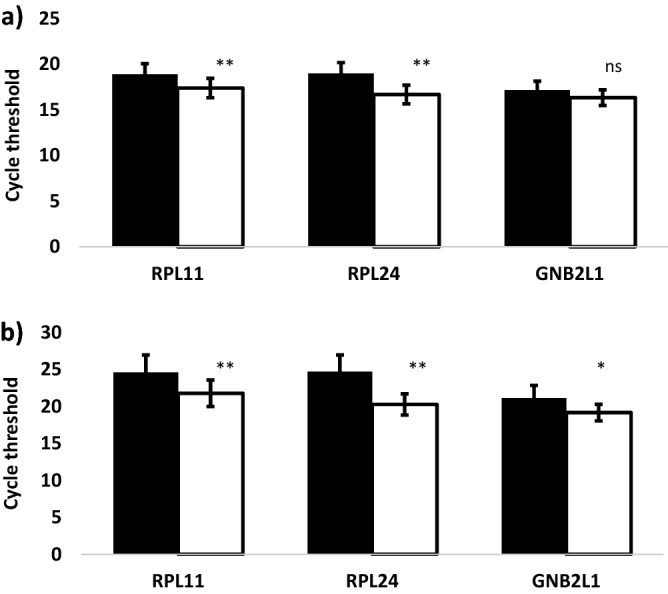
Benefit of selecting shorter probes for candidate endogenous control genes. Histograms show the mean ± SD of cycle threshold (Ct) values and the x-axes show the genes. (Long probes (solid columns) RPL11 142 bp; RPL24 156 bp; GNB2L1 75 bp. Short probes (empty columns) RPL11 106 bp; RPL24 86 bp; GNB2L1 66 bp). Asterisks indicate the level of significance for differences in Ct values by probe length (*P < 0.05; **P < 0.01; ns not significant). (**a**) *RPL11* (*p* = 0.0056), *RPL24* (*p* = 0.00086) and *GNB2L1* (*p* = 0.76) assayed in 12 prospective samples. (**b**) *RPL11 (p* = *0.0058), RPL24 (P* = *0.00000228)* and *GNB2L1 (p* = *0.00054)* assayed in 16 retrospective samples.

Table 2 lists the mean, SD and CoV of the Ct values for each of the 23 (16 on the TLDA array card and 7 single assay) gene probes assayed in the prospective and retrospective patient cohorts. The mean Ct values for all 23 probes were significantly lower when assayed in the prospective compared with the retrospective cohort (*p* < 0.0001). Figure 1 shows the mean Ct values for two probes of different lengths for *RPL11*, *RPL24* and *GNB2L1*. Ct values were significantly higher (significance levels indicated in Fig. 1) with the longer probes, except for GNB2L1 in the prospective samples. Shorter length gene probes for these genes were selected for subsequent analyses.

**Table 2:** Comparison of inter-sample gene expression in the prospective and retrospective cohorts. Mean (Ct), inter-sample SD and inter-sample CoV in gene expression (Ct) in FFPE tissue from prospective (12 samples) and retrospective (16 samples) bladder cancer cohorts.

Gene	Prospective samples (n = 12)			Retrospective samples (n = 16)			*P* value*
	Mean Ct	SD	CoV (%)	Mean Ct	SD	CoV (%)	
TaqMan™ endogenous control array							
*`18S rRNA*	23.29	1.71	7.60	30.43	2.39	7.86	4.60E−07
*ACTB*	22.09	1.26	5.71	29.50	4.85	16.47	9.20E−06
*B2M*	16.27	0.75	4.62	20.11	1.56	7.80	6.57E−08
*GAPDH*	18.51	1.19	6.44	23.44	2.18	9.29	7.53E−07
*GUSB*	20.15	0.86	4.25	24.06	1.56	6.49	2.63E−07
*HMBS*	23.08	0.96	4.18	25.58	1.21	4.71	2.95E−06
*HPRT1*	22.35	1.23	5.49	29.61	5.36	18.09	9.17E−07
*IPO8*	20.48	0.71	3.45	23.87	1.12	4.70	6.57E−08
*PGK1*	17.31	0.82	4.75	20.80	1.44	6.95	7.89E−07
*POLR2A*	19.37	0.70	3.59	21.99	0.80	3.64	6.57E−08
*PPIA*	16.71	1.10	6.59	20.38	1.59	7.78	2.63E−07
*RPLP0*	18.15	1.10	6.02	22.59	1.94	8.60	1.31E−07
*TBP*	25.57	1.13	4.40	31.92	4.16	13.03	9.17E−06
*TFRC*	20.40	1.20	5.88	23.85	1.50	6.28	7.89E−07
*UBC*	16.94	0.88	5.24	20.35	1.18	5.80	6.57E−08
*YWHAZ*	21.90	0.98	4.49	24.99	1.57	6.29	2.63E−07
Single tube assay							
*SDHA* (70)	20.18	0.81	4.00	23.00	1.37	6.00	1.28E−05
*RPL11* (142)	18.90	1.17	6.17	24.53	2.50	10.2	2.63E−07
*RPL11* (106)	17.40	1.06	6.09	21.91	1.91	8.70	2.63E−07
*RPL24* (156)	19.00	1.19	6.30	24.93	2.43	9.75	1.32E−07
*RPL24* (86)	16.69	1.02	5.78	20.26	1.52	7.47	2.63E−07
*GNB2L1* (75)	17.17	0.99	5.24	21.50	1.69	7.86	6.57E−08
*GNB2L1* (66)	16.35	0.86	6.11	19.23	1.15	5.98	2.63E−07

*The *p* values are from a Mann Whitney test comparison of mean Ct values in the prospective and retrospective data.

### Gene stability

**Figure 2:**
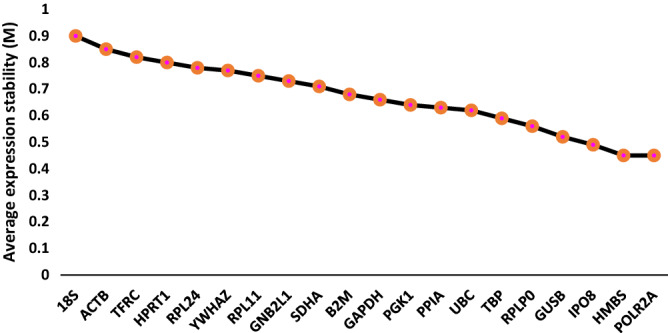
Plot of average expression stability values (M) of remaining candidate endogenous genes during stepwise removal of the gene least stable gene by GeNorm. Data are for 12 prospective samples and the order of the genes on the x-axis indicate their ranking with the least stable on the left. Successful exclusion of the least stable gene by determining the expression ratios of each gene paired with each of the other genes leads to a combination of the two most stably constitutively expressed genes (in this case HMBS and POLR2A).

**Figure 3:**
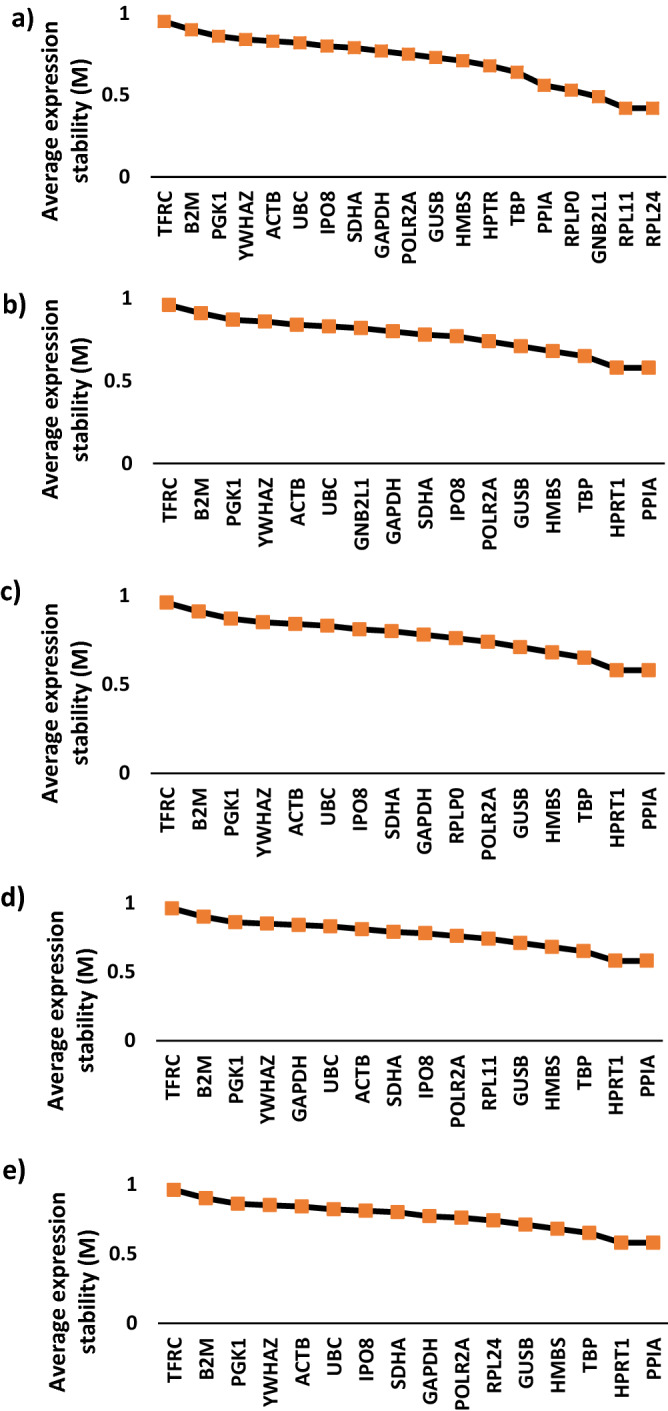
Influence of co-expressed genes on apparent gene expression stability. Plot of average expression stability values (M) of remaining candidate endogenous genes during stepwise removal of least stable genes by GeNorm based on TCGA sample cohort. All candidate endogenous control genes present in the analysis (**a**) excluding RPLP0, RPL11 and RPL24 (**b**) excluding GNB2L1, RPL11 and RPL24 (**c**) excluding GNB2L1, RPLP0 and RPL24 (**d**) excluding GNB2L1, RPLP0 and RPL11 (**e**).

Table 3 lists the candidate endogenous genes by stability as determined by CoV, GeNorm and NormFinder in the prospective (n = 12) and TCGA (n = 401) bladder cohorts. Figures 2 and 3a plot the candidate endogenous gene stability ranking by GeNorm in the prospective and TCGA cohorts respectively. GeNorm also defines the stability value for the combination of the two most stable genes. All candidate genes were expressed stably with values below the recommended M = 1.5 cut-off. In the prospective cohort GeNorm identified the combination of *SDHA* and *IPO8* as the most stable. Five genes (*UBC, RPLP0, HMBS, GUSB, and TBP*) were present in all the ten most stable genes ranked by CoV, GeNorm and NormFinder. In the TCGA cohort *PPIA* and *TBP* had the greatest stability by both CoV and NormFinder. However, GeNorm ranked the four co-expressed genes *RPLP0, RPL11, RPL24* and *GNB2L1* (Fig. 3) as exhibiting the most stable expression. Interestingly the next two genes were *PPIA* and *TBP*.

**Table 3:** Gene stability ranking: The ten most stable genes selected on the basis of lowest CoVs, or by inputting Ct values into GeNorm and Normfinder algorithms from the prospective bladder cancer cohort and TCGA bladder cancer cohort. (*’common’ refers to genes which appear in the top ten most stable genes in all three measures of stability (CoV, GeNorm and Normfinder)).

Rank		CoV		GeNorm	NormFinder			Common*
Prospective bladder cancer cohort								
1		*SDHA*		*SDHA*	*HMBS*			*UBC*
2		*IPO8*		*IPO8*	*PGK1*			*RPLP0*
3		*POLR2A*		*UBC*	*UBC*			*HMBS*
4		*HMBS*		*GUSB*	*GAPDH*			*GUSB*
5		*GUSB*		*RPLP0*	*PPIA*			*TBP*
6		*TBP*		*HMBS*	*GUSB*			
7		*UBC*		*PPIA*	*TBP*			
8		*RPLP0*		*RPL11*	*GNB2L1*			
9		*HPRT1*		*GAPDH*	*RPLP0*			
10		*YMHAZ*		*TBP*	*HPRT1*			
TCGA bladder cancer cohort								
1	*TBP*		*RPL11*			*PPIA*	*TBP*	
2	*PPIA*		*RPL24*			*TBP*	*PPIA*	
3	*IPO8*		*GNB2L1*			*UBC*	*HMBS*	
4	*UBC*		*RPLP0*			*IPO8*	*RPL11*	
5	*POLR2A*		*PPIA*			*RPL11*		
6	*RPL11*		*TBP*			*HMBS*		
7	*ACTB*		*HPRT1*			*SDHA*		
8	*YWHAZ*		*HMBS*			*GAPDH*		
9	*HMBS*		*GUSB*			*RPLP0*		
10	*RPL24*		*POLR2A*			*HPRT1*

To explore the possibility of bias due to co-expression GeNorm analysis was carried out omitting 3 of the 4 co-expressed genes. Figure 3 shows that when 3 of the 4 co-expressed genes were excluded from the analysis, expression levels of *PPIA* and *HPRT1* were the most stable with *TBP* and *HMBS* in third and fourth place. Co-expression accounts for some of the apparent high stability of these four genes when analysed collectively alongside all candidate genes by GeNorm. However, when analysed in the absence of co-expressing genes, *RPL11* and *RPL24* rank sixth, *RPLP0* seventh and *GNB2L1* tenth suggesting that their expression is sufficiently stable to use as endogenous controls.

### Assessing the performance of the selected endogenous control genes

**Figure 4:**
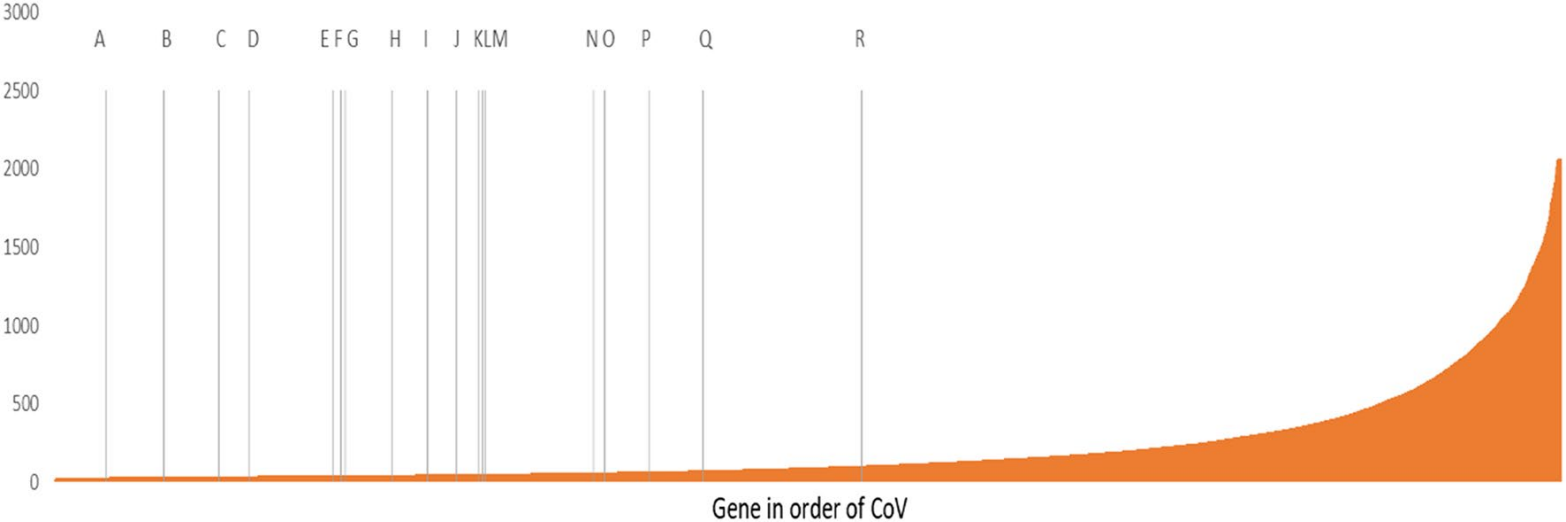
Plot of gene expression CoV for TCGA bladder cancer cohort (n = 401 samples) highlighting candidate endogenous control gene panel: TBP (**A**), PPIA (**B**), IPO8 (**C**), UBC (**D**), POLR2A (**E**), RPL11 (**F**), ACTB (**G**), YMHAZ (**H**), HMBS (**I**), RPL24 (**J**), GNB2L1 (**K**), RPLP0 (**L**), GAPDH (**M**), PGK1 (**N**), GUSB (**O**), HPRT1 (**P**), B2B (**Q**), TFRC (**R**).

**Figure 5:**
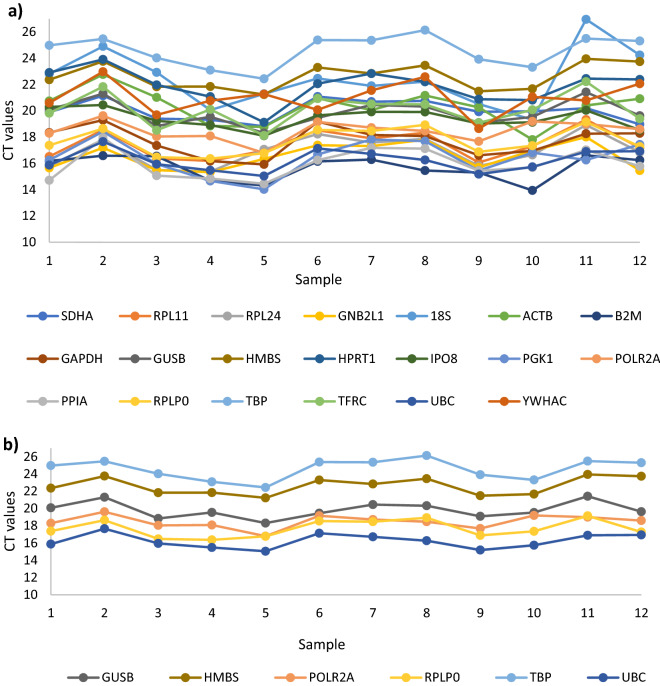
Co-ordination between candidate endogenous control gene expression in prospective bladder cancer samples. All candidate endogenous control genes (**a**) and genes ranked in the ten most stable by CoV, GeNorm and Normfinder (**b**).

Genes in the bladder cancer cohort extracted from the TCGA were ordered by CoV and the candidate endogenous genes highlighted (Fig. 4). All the candidate endogenous control genes fell within the lower 50% of CoV values. The most stable seven genes (*TBP, PPIA, UBC, IPO8, POLR2A, RPL11 and ACTB*) are within the lowest 20% CoV values. Figure 5 shows the most stably expressed endogenous control genes have coordinated changes in Ct values when assayed in different samples showing they are influenced similarly by differences in RNA quality, reverse transcription efficiencies and other factors associated with sample preparation.

## Discussion

Measuring gene expression is increasingly important for a diverse range of clinical applications^23–26^. Purification of RNA, an essential prerequisite for qPCR, removes other cellular components and data must be normalised based on the stable expression of endogenous control genes. Analysis of gene expression studies showed using a single endogenous control gene^11^ can produce gene expression error values of over 20-fold suggesting that multiple endogenous control genes are required for normalisation.

Selection of endogenous control genes needs to be rigorous and take account of potential confounding factors which may be study specific. Commonly used endogenous control genes shown to be stable in one tissue type and set of conditions may be unsuited for others. *GAPDH* and β-actin and suitable for qPCR normalisation in some situations because they are expressed at high and constant levels in many cells and tissues^27,28^. However, a study using *GAPDH* and *ACTB* as endogenous control genes demonstrated aberrations in qPCR results due to the regulatory effects of microRNAs on the expression of these genes^7^. Further, the expression of *GAPDH* correlates highly with *CA9*, a marker of hypoxia, precluding the use of *GAPDH* as an endogenous control gene for studies involving solid tumours.

**Figure 6:**
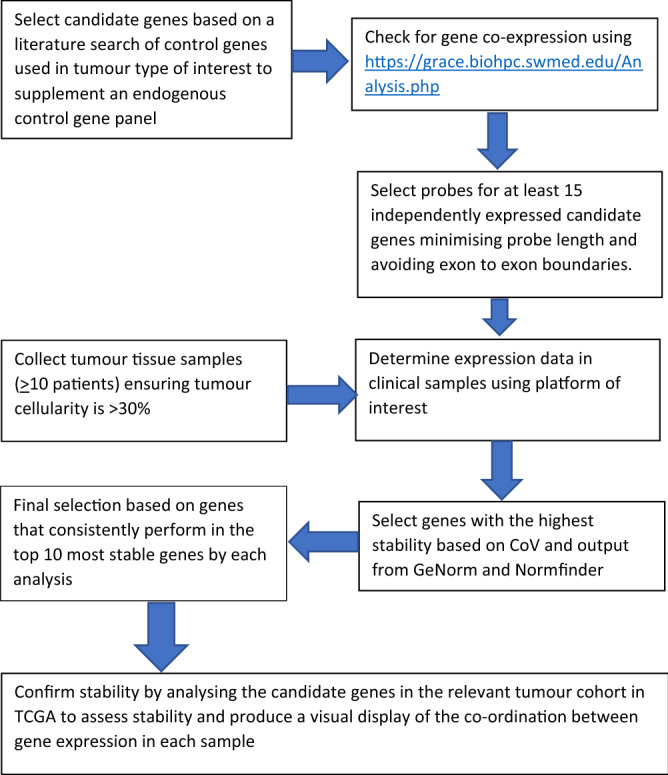
Steps involved in selection of endogenous control genes for normalisation of FFPE tumour tissue.

In this study we have described a workflow that uses a combination of laboratory and software tools to select a set of endogenous control genes for qPCR studies. The protocol is summarised in Fig. 6.

Both GeNorm and Normfinder can identify the most stable from the least stable endogenous control genes^29^. However, in common with other studies^30,31^ the order of gene stability ranking by the two algorithms differed for both the prospective and TGCA cohorts. GeNorm uses pairwise comparison of candidate endogenous control genes to test for gene expression stability^11^ to stepwise eliminate the least stable genes. NormFinder is a mathematical model based on ANOVA which calculates an overall average expression level for all genes to which it compares the expression of each individual gene and ranking according to stability^12^. For small sample size studies GeNorm is recognised as the more reliable algorithm for determining gene expression stability^29^. On the other hand, Normfinder is considered more robust than GeNorm for studies with larger sample numbers. GeNorm can preferentially select genes that are coregulated which mutually reinforce and so bias the apparent expression stability of co-expressed genes.

Using GeNorm, *POLR2A* and *HMBS* were identified as the most stable gene combination in the prospective sample cohort. GeNorm but not Normfinder ranked the four co-expressed genes as the most stable of the candidate group when analysed in the TCGA cohort. To test the possibility that GeNorm was selecting these genes due to bias through co-expression the analysis was repeated excluding 3 of the 4 co-expressed genes in our endogenous control gene panel. In each case the remaining gene remained within the top ten genes but ranked lower. This finding suggests that these genes can still be used for normalisation but when analysed together using GeNorm multiple co-expressed genes can provide mutual reinforcement in stability score which overstates their actual stability, at least in part, explaining the difference of the overall endogenous control ranking by GeNorm and Normfinder.

Interestingly GeNorm did not rank the co-expressed genes highly in the prospective muscle invasive bladder cancer cohort. Overall ranking of the endogenous control genes by both GeNorm and Normfinder differ between the two cohorts. These different rankings are not surprising as gene expression data in TGCA is acquired using RNAseq and normalised. Gene expression data in the TCGA is also acquired using RNA extracted from fresh-frozen tissue which will be less modified than that from FFPE. However, it has been shown that RNA expression acquired using FFPE maintains the fidelity of patterns in biological signals and relationships with patient outcomes consistent with studies using fresh-frozen tissue^32^.

Taqman PCR gene expression methodology requires complete hybridisation of gene probes to cDNA sequences to register a hit which would suggest that shorter gene probe sequences will improve gene expression detection especially in degraded samples. Consistently Ct values were found to be significantly lower compared with long probes when using shorter gene probes for *RPL11, RPL254* and *GNB2L1* demonstrating the importance of choosing shorter length probes to reduce the risk of sample gene dropout especially when using archived FFPE samples.

In summary, our work highlights the importance of probe length and the need to account for co-expression when selecting a panel of endogenous control genes for downstream application in clinical samples. We identified a set of six genes that are stably expressed in FFPE bladder cancer samples and are suitable for use as endogenous control genes. We also recommend use of our workflow to harmonise the process of endogenous control selection qPCR-based studies.
